# Salivary phosphate as a biomarker for human diseases

**DOI:** 10.1096/fba.2021-00104

**Published:** 2022-01-03

**Authors:** Mohammed S. Razzaque

**Affiliations:** ^1^ Department of Pathology Lake Erie College of Osteopathic Medicine Erie Pennsylvania USA

**Keywords:** biomarker, obesity, phosphate homeostasis, saliva

## Abstract

Phosphate is a common ingredient of the daily consumed foods and is absorbed in the intestine and is excreted in the urine through the kidney to maintain the homeostatic balance. For adults, the Recommended Dietary Allowance (RDA) for phosphorus is around 700 mg/day. The change in dietary habits resulted in far more phosphate consumption (almost double) than the RDA, contributing to increased cardiovascular diseases, kidney diseases, and tumor formation. Due to a lack of clinical appreciation for the long‐term consequences of chronic phosphate burden on non‐communicable disorders, it is rapidly becoming a global health concern. The possible association between dysregulated phosphate metabolism and obesity is not studied in‐depth, mainly because such an association is believed to be nonexistent. However, in the animal model of obesity, serum phosphate level was higher than their non‐obese controls. In a similar observation line, significantly higher salivary phosphate levels were detected in obese children compared to normal‐weight children. Of clinical importance, despite the significant increase of salivary phosphate levels in obese children, the plasma phosphate levels did not change in samples collected from the same group of children. Such disparity between plasma and saliva raised the possibility that human salivary phosphate levels may be an early biomarker of childhood obesity.

## 1 INTRODUCTION

Phosphorus is an essential nutrient that plays crucial roles in the synthesis of DNA and RNA (as nucleic acid), energy metabolism (as ATP), cellular activation, and signaling (as protein phosphorylation), and the maintenance of membrane integrity (as phospholipids).^1^ Phosphorus is a natural element, exclusively present in the phosphorus‐containing compound phosphate. After consuming phosphorus through food, it is oxidized into phosphate within the body. The term phosphate (PO_4_) will be used to describe this element throughout this article. The optimal level of phosphate is also critical for skeletal growth and function.^2^ The phosphate has a strong electronegative attraction with calcium ions. Approximately 85% of phosphate in the human body is either in the bones or teeth, as hydroxyapatite (calcium‐phosphate salt).^3^ The intestine absorbs the phosphate from the consumed food into the blood, while the kidneys maintain optimal serum phosphate concentration through reabsorption and urinary excretion. Bone cell‐derived fibroblast growth factor 23 (FGF23) actively regulates the renal reabsorption of filtrated phosphate.^4^, ^5^, ^6^

Genetically modified animal studies have shown that FGF23 can increase urinary excretion of phosphate by reducing renal reabsorption. For instance, FGF23 transgenic mice develop hypophosphatemia due to excessive urinary phosphate excretion.^7^, ^8^ In contrast, FGF23 knockout mice develop hyperphosphatemia, partly due to increased renal reabsorption of phosphate.^9^, ^10^ Elevated serum phosphate can bind with free calcium to form Calcium x Phosphate product, which is then deposited into the cardiovascular system to induce cardiovascular calcification. In contrast, inadequate phosphate consumption usually leads to skeletal mineralization defects in premature children or individuals with malnutrition (including chronic alcoholism). Additionally, impaired intestinal phosphate absorption or excessive renal phosphate wasting can cause hypophosphatemia to induce rickets. Reduced intestinal phosphate absorption can occur in patients with reduced vitamin D activity (either deficiency or resistance), individuals using oral phosphate binders, and patients suffering from steatorrhea (decreased absorption of fat‐soluble vitamin D).^11^ Excessive renal phosphate loss can occur in patients with hyperparathyroidism, patients with enhanced FGF23 activities (genetic diseases), or patients with inactivation of sodium‐dependent phosphate transporters in the kidneys.^12^, ^13^, ^14^, ^15^, ^16^ The normal serum phosphate levels in adults range from 2.4 to 4.5 mg/dl. Minor symptoms might appear when serum phosphate levels are around 1 to 2 mg/dl, but severe skeletal symptoms are likely to occur when serum phosphate level is down to 1mg/dL.^12^

On the other hand, phosphate overload is far more common and can cause various human diseases, such as gingivitis, dental decay, cardiovascular diseases, renal diseases, premature aging, and tumorigenesis.^17^, ^18^, ^19^, ^20^, ^21^ Elevated phosphate can adversely influence the cell behaviors of osteoblasts,^22^ osteoclasts,^23^ and vascular smooth muscle cells.^24^, ^25^, ^26^ In human lung cells, elevated phosphate can accelerate cell growth to potentiate tumorigenesis. Experimental studies conducted on genetically modified mice have shown that elevated phosphate propagated pulmonary tumors by inducing cell proliferation and angiogenesis.^27^ Recent research has shown that abnormally high phosphate concentrations can also cause cell death by necrosis and apoptosis.^28^ Genetically induced hyperphosphatemic mice resulted in severe structural alterations in the lung, heart, kidney, and blood vessels.^29^, ^30^, ^31^, ^32^, ^33^, ^34^ It appears likely that the detection of dysregulated phosphate levels might provide an early clue of eventual tissue and organ damages. Therefore, it has the potential to be a biomarker for various systemic and metabolic diseases.

Obesity is a global health problem and is often associated with dyslipidemia, cardiovascular diseases, metabolic diseases, and cancer.^35^ According to the World Health Organization's estimation, around 2.3 billion people are globally overweight, and more than 700 million people are obese. The potential effects of dysregulated phosphate metabolism and obesity have not been studied in‐depth and in detail, as the earlier studies have mainly focused on determining the roles and regulation of phosphate on skeletal diseases.^36^, ^37^, ^38^ Experimental animal studies, however, have shown that leptin‐deficient obese (ob/ob) mice have high serum phosphate levels as compared to their non‐obese wild‐type (WT) mice.^39^, ^40^ Similarly, in childhood obesity, salivary phosphate level was higher than the non‐obese children.^41^

## 2 SALIVA AS A BIOMATERIAL

**FIGURE 1:**
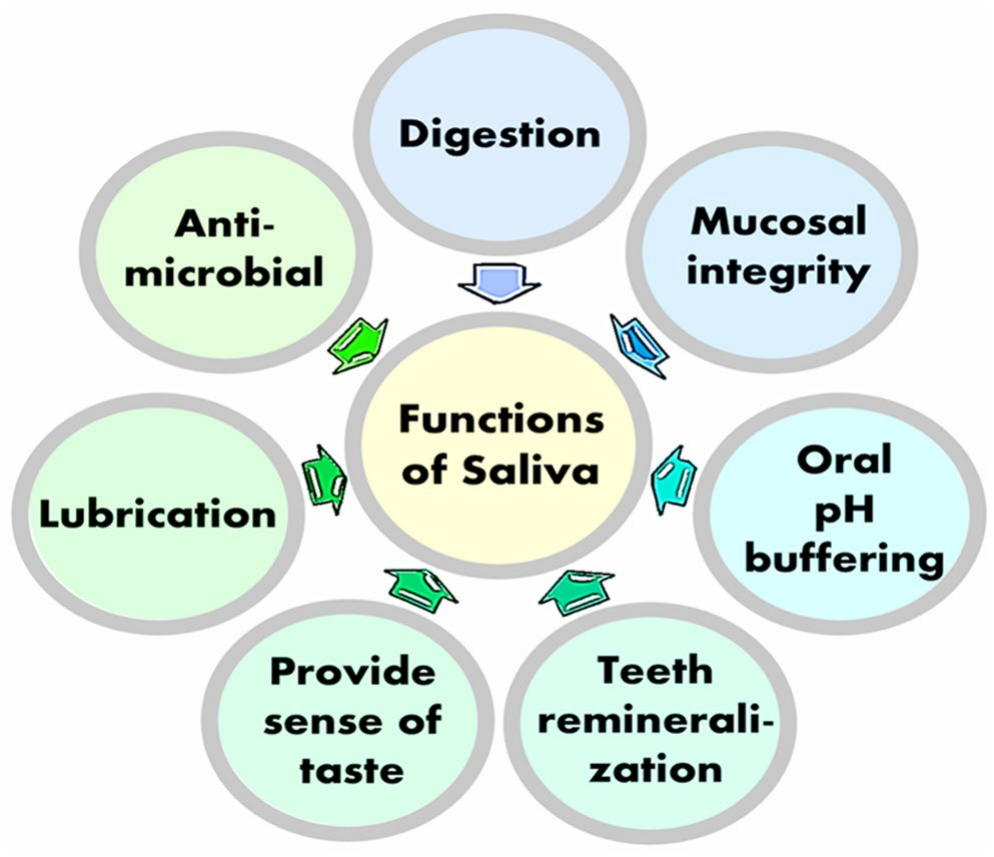
Main functions of saliva in oral cavity

Saliva, produced and secreted from the salivary glands, exerts numerous essential biological functions, ranging from maintaining optimal oral pH to providing antimicrobial defense to the oral cavity (Figure 1). Saliva is a noninvasively available biomaterial and can be effectively used to detect biomarkers, such as insulin, C‐reactive protein (CRP), and adiponectin in metabolic diseases.^41^ Moreover, salivary phosphate level was associated with inflammatory markers, including salivary CRP in oral diseases.^19^ Ghrelin, commonly known as the “hunger hormone”, can increase appetite, enhance food intake, and promote fat storage to contribute to the development of obesity. Of importance, the salivary glands can synthesize ghrelin.^42^, ^43^, ^44^ A higher concentration of ghrelin in saliva than in serum is also detected in obese individuals.^43^ In a similar line of observation, peptide YY, a satiety hormone, is detected in both murine and human saliva.^45^ A feeling of fullness can be accomplished by the acute increase in peptide YY in the saliva. The prolonged increase in salivary peptide YY caused a significant long‐term decrease in food consumption and reduced body weight in an experimental model of obesity.^45^

Currently, detecting the blood‐based biomarker is the gold standard for monitoring non‐communicable diseases, including obesity. Though blood‐based tests are essential for determining the spectrum of various diseases with clinical applications, ranging from screening to diagnosis to following up to predict the course of the disorders, blood‐based biomarker study methods are not always risk‐free. For instance, the risk of transmission of blood‐borne pathogens through contaminated sharp instruments or needles while collecting blood samples is a serious health concern, particularly in underdeveloped countries. Blood collection may also induce transient discomfort and bruising, and infection at the site of venipuncture. Moreover, healthcare workers have a higher accidental risk of contracting transmissible diseases such as human immunodeficiency virus (HIV), hepatitis C virus (HCV), and hepatitis B virus (HBV), while collecting blood.^46^ Studies conducted on healthcare workers have estimated that the average HIV transmission risk is approximately 0.3%, HCV transmission risk is approximately 1.8%, and HBV transmission risk is 6 to 30%.^47^ Of clinical importance, compared to the blood collection, saliva sample collection lowers the risk of infection transmission, with increased patient convenience. As mentioned, obesity is a significant risk factor for many systemic diseases, and some of those diseases are known to influence the salivary glands and oral health.^48^, ^49^ However, information on the salivary gland function during the commencement of obesity is limited. This article will briefly discuss the significance of elevated salivary phosphate levels as a potential biomarker of the evolvement of childhood obesity and other systemic diseases.

## 3 SALIVARY PHOSPHATE AND HUMAN DISEASES

### 3.1 Chronic kidney disease (CKD)

Studies have documented increased salivary phosphate levels in patients with CKD, with a linear association between serum and salivary phosphate levels.^50^, ^51^ The association of hyperphosphatemia and higher CKD patients’ mortality is well‐established in clinical settings.^52^, ^53^ In patients with CKD, hyperphosphatemia is also a surrogate marker of cardiovascular calcification and clinical outcomes, including all‐cause hospitalization and mortality.^52^, ^53^, ^54^ It is estimated that the salivary phosphate level is nearly doubled in patients with CKD.^50^ Of clinical importance, salivary phosphate levels positively correlate with serum creatinine and the glomerular filtration rate in patients with CKD.^51^ The presence of high creatinine and uric acid levels in the plasma and saliva of obese children also implies the possibility of association with renal diseases.^55^ Of clinical significance, reducing salivary phosphate content by using phosphate‐binders has been shown to reduce serum phosphate levels in patients with CKD undergoing hemodialysis treatment.^56^ Although further studies are needed, it appears likely that manipulating salivary phosphate might have therapeutic value in managing systemic diseases, including CKD.

### 3.2 Oral diseases

Salivary calcium and phosphate delicately work together to contribute to the tooth's demineralization and remineralization process to avert dental caries and erosions.^57^, ^58^ Loss of homeostatic balance of calcium and phosphate in the saliva can disrupt the mineralization process to increase the susceptibility of dental caries and periodontal diseases.^59^ The increased amount of phosphate consumption is associated with a higher dental decay rate in children.^19^ After adjusting all the covariates of 8317 studied children, the odds of having dental decay increased by 33% for every single unit increase in calorie‐adjusted phosphate intake.^19^ Similarly, after adjusting for age, sex, body mass index, and gingival redness, the daily dietary phosphate consumption was strongly correlated with the salivary level of IL‐1β; it was inversely correlated with the salivary level of IL‐4 in patients with gingivitis.^21^ The disproportionate salivary levels of IL‐1β and IL‐4 in patients with gingivitis are likely to be the molecular signature of oral inflammation, possibly induced by increasing phosphate consumption.^21^ Again, the accumulating evidence is signifying the importance of salivary markers in diagnostic and follow‐up purposes.

### 3.3 Childhood obesity

**FIGURE 2:**
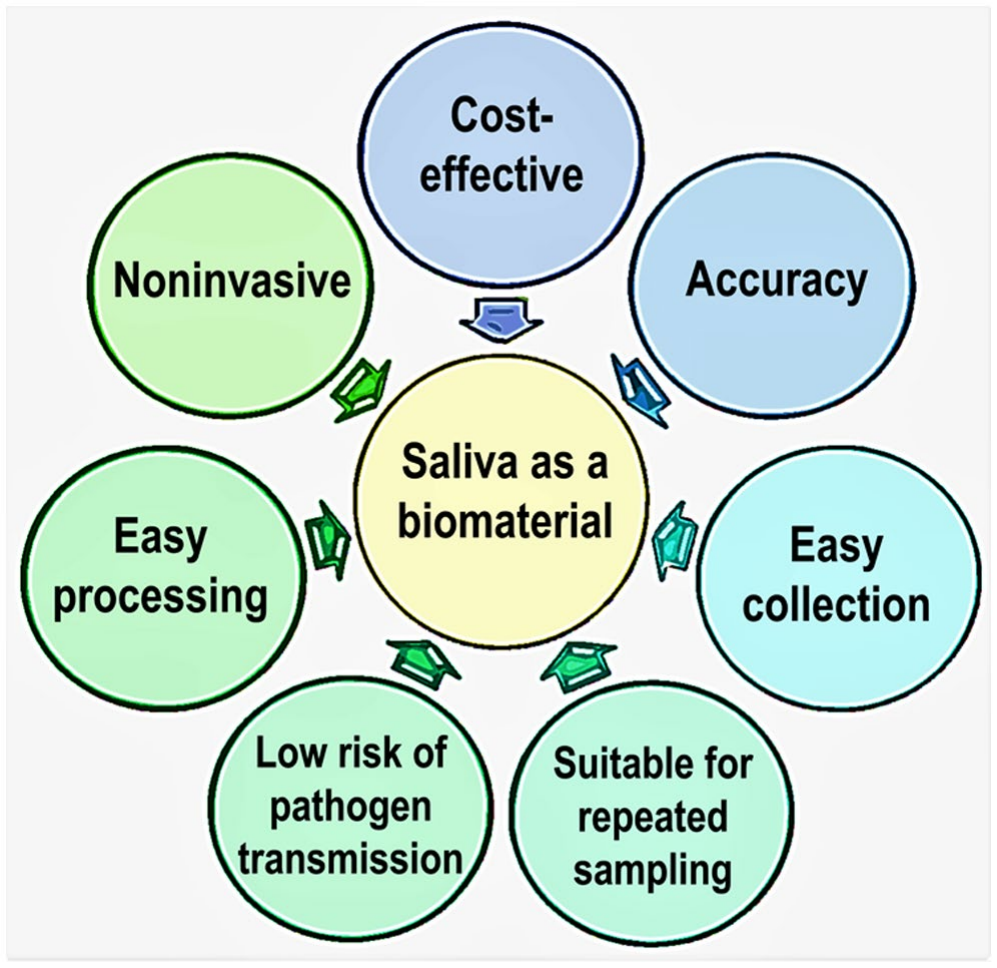
Main advantages of saliva as a biometerial over blood‐based tests

**FIGURE 3:**
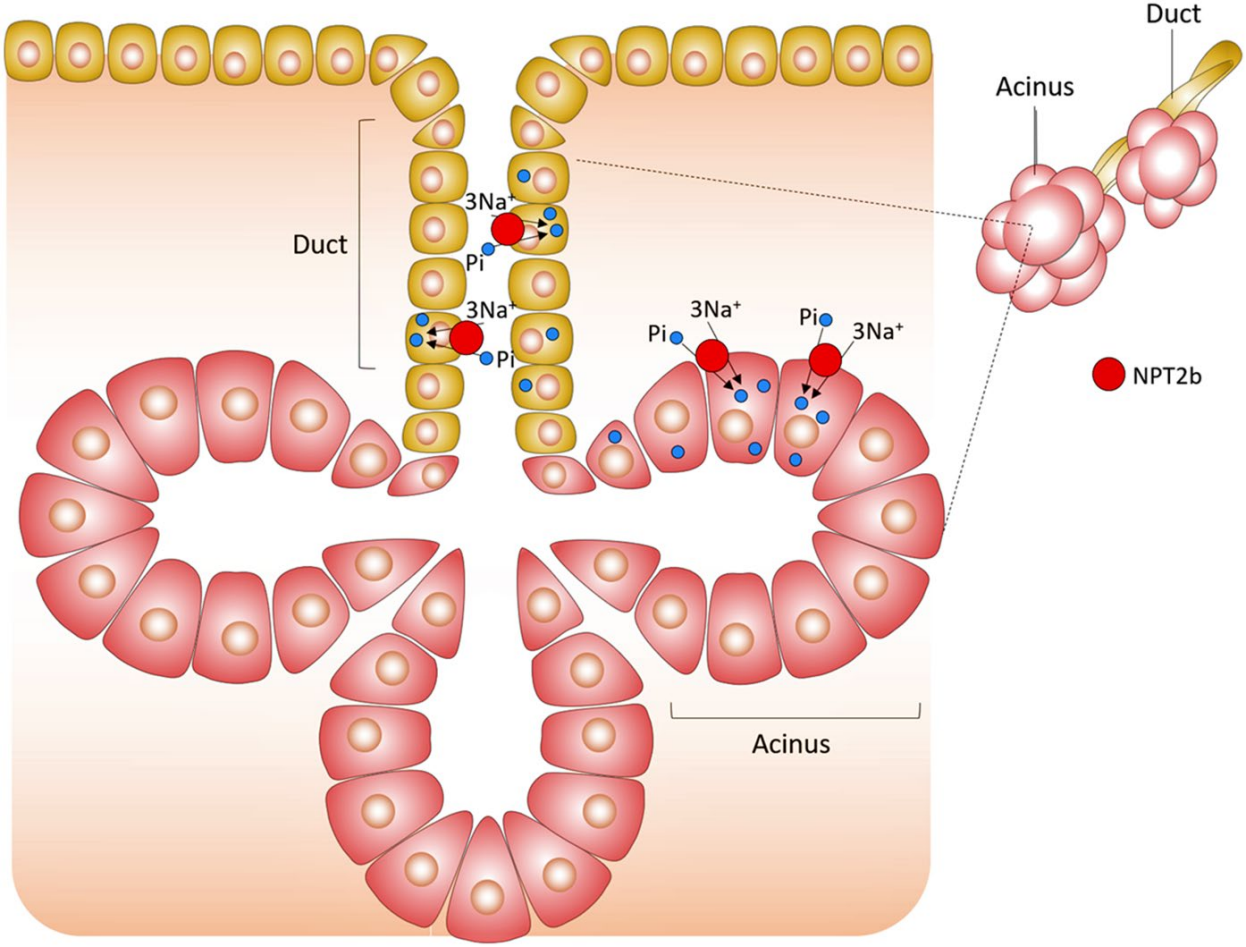
A schematic diagram shows the presence of type II sodium phosphate cotransporters (NPT2b) in the acini and ducts of the salivary gland. In acinar cells, NPT2b is present primarily in the basal–lateral area. In contrast, in duct cells, NPT2b is present in the apical side of the cells. NTP2b activities are likely to regulate phosphate secretion and reabsorption in the salivary glands to control the concentration of phosphate in the saliva^63^

Because of the non‐invasive sample collection, saliva has a significant advantage over peripheral blood, particularly for screening the disease development in children (Figure 2). Adiponectin is a product of fat cells and can exert anti‐inflammatory effects.^60^ An inverse association of salivary level of adiponectin and the occurrence of childhood obesity has also been reported. In a studied cohort of 744 children, the salivary CRP insulin, and leptin levels were 3 to 6 times more elevated in obese children than their lean counterparts; adiponectin was 30% lower in obese children than healthy normal‐weight children.^41^ Of significance, salivary insulin level predicting hyperinsulinemia was found in 4.3% of normal‐weight adolescents, 8.3% of overweight adolescents, and 25.7% of obese adolescents (*p* < 0.0001), while salivary glucose predicting hyperglycemia was detected in only 3% of obese children and was not statistically significant (*p* = 0.89).^61^ Simultaneous analysis of phosphate in saliva and plasma collected from 77 children had shown a significant (ANOVA *p* < 0.001) increase of salivary phosphate level detected in obese children compared to normal‐weight children. Despite the elevated salivary phosphate content in obese children, the level of phosphate in plasma was normal in children's same cohort.^62^ The cause for the higher salivary phosphate concentration in obese children is not fully known. However, it is necessary to mention that the molecular machinery (FGF23, klotho, FGF receptors, type II and type III sodium phosphate cotransporters) involved in regulating renal phosphate homeostasis are also present in the salivary glands^63^ (Figure 3). The possibility of salivary regulation of phosphate that is independent of systemic regulation might explain why salivary phosphate is higher in obese children without affecting plasma phosphate level.^62^ Further studies will be needed to understand such disparity between blood and salivary phosphate in obese children. From the clinical perspective, significantly altered salivary phosphate levels and other obesity‐associated biomarkers suggest a potential for developing non‐invasive screening systems to identify the vulnerable children at risk of developing obesity for early intervention and preventative care.

Despite being an easily collectible biomaterial, and more than 5000 proteins are identified in saliva,^64^ due to low specificity, lack of validation studies, and the absence of comparative data of saliva‐ and blood‐based tests limit the widespread clinical use of saliva as a biomaterial. Interpretation of saliva assays is challenging, as standardization of normal salivary ranges for various biomolecules has not yet been established or validated through large cohort measurements; verifying the sensitivity and specificity of salivary analysis needs further studies. When CRP level was measured both in saliva and serum of adult individuals, a dichotomous index of salivary CRP was detected equivalent to serum levels of CRP.^65^ Similarly, compared to the healthy controls, a highly significant correlation (*p* < 0.001) was noted between the salivary glucose and serum glucose levels in diabetic patients.^66^ Despite no significant changes in serum phosphate levels between healthy adult controls (52.3 ± 8 years) and diabetic patients (48.3 ± 7 years), the salivary phosphate level was significantly (*p* < 0.01) higher in diabetic patients (*n* = 60; 13.7 ± 4.4 mg/dl) as compared to the healthy controls (*n* = 60; 8.3 ± 1.9 mg/dl).^66^ This shows a trend of elevation of salivary phosphate levels, even before apparent changes in serum level of phosphate in metabolic diseases. Recently, a comparative genotyping study obtained identical results for DNA collected from salivary material and blood samples by high throughput genotyping.^67^

## 4 CONCLUSIONS

Accrued evidence suggests a biological basis to consider that abnormal phosphate regulation could adversely impact the routine oral functions by promoting oral and systemic inflammation.^19^, ^21^ Of relevance, inflammation is an active pathomechanism of obesity.^68^ As mentioned, CRP levels (a sensitive marker of systemic inflammation) in obese children's saliva were significantly higher than those in their normal‐weight counterparts.^41^, ^69^ Studies have also found disease reciprocity between gingivitis and obesity. Obesity could augment gingivitis, while gingivitis could also enhance obesity by activating neutrophils and other inflammatory cells to create a pro‐inflammatory microenvironment to enhance adipogenesis.^55^, ^70^ Moreover, the increased salivary phosphate levels before detectable changes in blood could help identify the population at risk of evolving childhood obesity or monitor the progression of childhood obesity. The significance of using salivary biomarkers as part of a patient screening system to diminish blood contamination‐related disease transmission and to make it more cost‐effective, without affecting the quality of health care is a growing area of clinical research. Finally, salivary phosphate level may be used for community screening to identify children at risk of developing childhood obesity and has the potential to be a critical non‐invasive predictive marker in the development of obesity. Additional studies will illuminate the underlying mechanism of why salivary phosphate level is high in obese and overweight children. As mentioned, the elevation of salivary phosphate even before systemic changes of phosphate in the blood could be of diagnostic value, particularly in monitoring the progression of obesity. Finally, the use of salivary biomarkers in community screening and longitudinal studies to investigate the evolvement of childhood obesity in metabolic diseases could result in early intervention to reduce disease burden.
